# Renal function affects the plasma concentration and hematological toxicity of linezolid in elderly patients: a propensity-matched cohort analysis

**DOI:** 10.1186/s12877-025-06241-9

**Published:** 2025-08-06

**Authors:** Bing Liu, Guangrui Geng, Tingting Liu, Lingli Zhou, Fang Wang, Ping Yang, Jiaxin Liu, Junshuai Zhao, Cheng Zhao, Yue Wang, Minna Yang, Jing Fu, Jingyue Chen, Xiaomin Chen, Xiangqun Fang, Lixin Xie, Hongxia Li

**Affiliations:** 1 Department of Pulmonary and Critical Care Medicine, The Second Medical Center and National Clinical Research Center for Geriatric Diseases, Chinese PLA General Hospital, Beijing, 100853 China; 2 Department of adult cardiac surgery, Sixth Medical Center of Chinese PLA General Hospital, Beijing, 100853 China; 3 Department of Critical Care Medicine, 920th Hospital of Joint Logistics Support Force of Chinese PLA, Kunming, Yunnan Province 650032 China; 4 BGI Genomics Co., Ltd, Shenzhen, 518083 China; 5 College of Pulmonary and Critical Care Medicine, 8th Medical Center of Chinese PLA General Hospital, Beijing, 100091 China

**Keywords:** Linezolid, Elderly patients, Renal function, Trough concentration, Hematological toxicity

## Abstract

**Background:**

The relationships of renal function with linezolid trough concentration (C_min_) and hematological toxicity have not been clarified in elderly patients.

**Methods:**

In this propensity-matched cohort analysis, elderly patients receiving linezolid at four tertiary hospitals in Beijing between May 2021 and March 2024 were included. The patients were grouped by renal function and propensity score matching (PSM) was used to balance baseline characteristics. Linezolid C_min_ and hematological indices were monitored dynamically.

**Results:**

Among 320 patients, 152 were eventually enrolled after PSM, including 56 (36.8%) patients with normal renal function (RN) and 96 (63.2%) patients with renal dysfunction (RD). C_min_ at 3–5 (12.5 mg/L vs. 21.2 mg/L, *P* = 0.001) and 6–10 days (14.8 mg/L vs. 27.4 mg/L, *P* < 0001) was significantly lower in the RN group. The risk of linezolid-associated thrombocytopenia (LAT) and linezolid-associated anemia (LAA) in the RD group was 4.485 folds and 2.308 folds higher than in the RN group. The decreases in hematological indices were significantly higher in RD group than in RN group.

**Conclusions:**

Renal function significantly affected linezolid C_min_ and hematological toxicity, and the dose of linezolid should be reduced in elderly patients with renal dysfunction.

**Trial registration:**

ChiCTR2100045707; Registration Date: 23/04/2021.

## Introduction

Linezolid is an oxazolidinone antibiotic with a broad spectrum of activity against gram-positive bacteria, including methicillin-resistant *Staphylococcus aureus* (MRSA) and vancomycin-resistant *Enterococcus* [1]. The efficacy of linezolid is mainly linked to the ratio of the area under the concentration–time curve (AUC) over 24 h to the minimum inhibitory concentration (MIC) [1]. Linezolid trough concentration (C_min_) has a significant linear relationship with AUC, and the incidence of linezolid-associated thrombocytopenia (LAT) increases significantly when C_min_ exceeds 8 mg/L. Therefore, the recommended target for the C_min_ of linezolid is 2–8 mg/L [2, 3].

According to the manufacturer’s instructions, the dose of linezolid does not need to be adjusted when administered to elderly patients. However, a growing number of studies have reported linezolid overuse in elderly patients [4, 5], and the recent Chinese expert consensus on linezolid recommended therapeutic drug monitoring (TDM) in elderly patients treated with linezolid to avoid overexposure [6]. Our previous study revealed that linezolid C_min_ was dramatically higher in the elderly, reaching approximately 10 mg/L in patients aged 65–80 years, followed by a further increase of 10 mg/L for every additional 10 years of age [7]. Therefore, it is urgent to dynamically monitor C_min_ and adjust the dose of linezolid in the elderly population. However, dynamic monitoring of linezolid C_min_ is not available in many institutions, making it essential to identify risk factors for elevated C_min_. A series of studies revealed that linezolid C_min_ is closely related to renal function [3, 8–13]. However, in the elderly population, the correlation between renal function and linezolid C_min_ has not been fully clarified.

The most common side effect of linezolid is hematological toxicity, especially thrombocytopenia, which occurs in 7.5–64.7% of patients and affects prognosis [3]. A significant increase in the incidence of LAT has been reported in non-elderly patients with renal dysfunction [14–19]. However, few studies have explored the effect of renal function on linezolid-associated hematological toxicity in the elderly population.

In this propensity-matched cohort analysis, we prospectively included elderly patients receiving linezolid, and we dynamically monitored linezolid C_min_ and hematologic indices, analyzed the effects of renal function on linezolid C_min_ and hematological toxicity.

## Materials and methods

### Patients

**Fig. 1:**
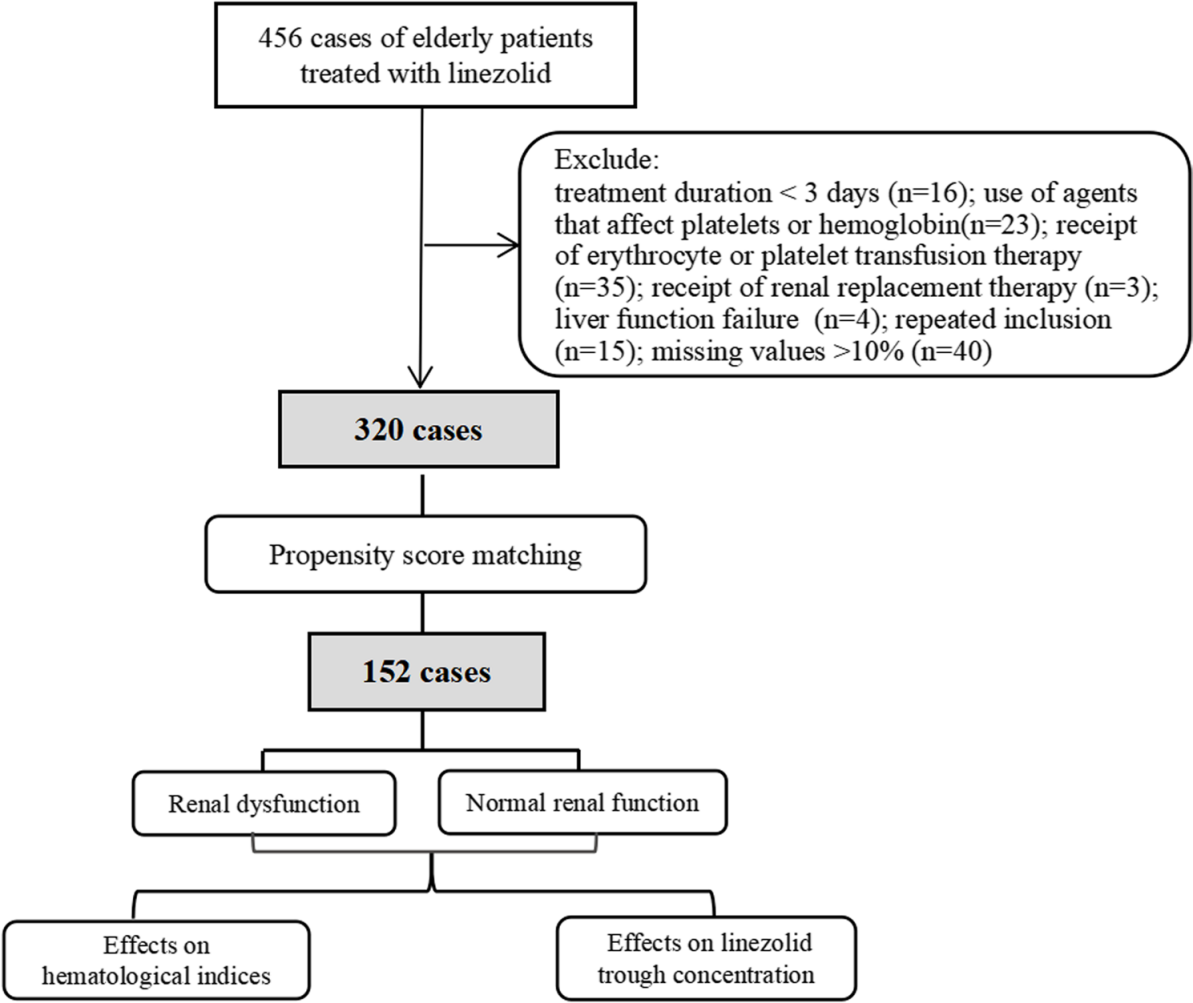
Flow chart of patients enrollment and study design

Elderly patients (≥ 65 years old) treated with linezolid at the First Medical Center, Second Medical Center, Fourth Medical Center, and Eighth Medical Center of the Chinese PLA General Hospital from May 2021 to March 2024 were included. The exclusion criteria were as follows: treatment duration < 3 days; use of agents that affect platelets or hemoglobin (e.g., chemotherapy drugs); receipt of erythrocyte or platelet transfusion therapy; receipt of renal replacement therapy; liver function failure (Child-Pugh score ≥ 10); repeated inclusion; and missing *values* > 10% (Fig. 1).

### Data collection

Basic information on the subjects included sex, age, underlying diseases, infection sites, the Charlson comorbidity index (CCI), the Sequential Organ Failure Assessment (SOFA) score, laboratory findings, the estimated glomerular filtration rate (eGFR), and prognosis.

### TDM of linezolid

The patients were treated with 1200 mg of linezolid (Zyvox, Pfizer) per day (600 mg twice daily) by either the intravenous or oral route. In total, 95.3% (305/320) of the patients adopted an alternating regimen of one intravenous and one oral administration, and 4.7% (15/320) of the patients used all lineazolid tablets. As a non-intervention study, clinicians independently decided to withdraw linezolid based on the clinical treatment effect and hematological indices. The C_min_ of linezolid was detected using liquid chromatography–tandem mass spectrometry (LC–MS/MS) [20], which had a linear detection range of 0.8–100 mg/L and a lower limit of quantification of 0.8 mg/L. The observed intra- and interday assay imprecision and inaccuracy were < 10%.

At 3–5 and 6–10 days after the first dose, venous blood samples were collected immediately before the subsequent dose of linezolid to assess C_min_, which was recorded as C_min,3−5d_ and C_min,6−10d_. In a real-world study, some patients refused to have their blood taken frequently. Thus, a secondary C_min_ measurement was not performed in all patients. The average trough concentration (C_ave_) of linezolid was defined as the average C_min_ during treatment. If the patient had only one C_min_ measurement, that value was the C_ave_. The distributions of linezolid C_min_ were defined as < 8 mg/L, 8–16 mg/L, 16–32 mg/L, and > 32 mg/L.

### Definitions of hematological toxicity and eGFR

Platelet and hemoglobin counts were monitored dynamically during linezolid therapy, especially at baseline, on the day of drug withdrawal, and at 3 day after drug withdrawal. Thrombocytopenia was defined as a decrease in the platelet count of > 30% versus baseline after the initiation of linezolid treatment to 72 h after withdrawal [3, 7, 10]. Decreases of 30–49%, 50–69%, and > 70% were defined as mild, moderate, and severe, respectively [7]. Anemia was defined as a decrease in the hemoglobin level of > 10% versus baseline after the initiation of linezolid treatment to 72 h after withdrawal [7]. Decreases of 10–19%, 20–29%, and ≥ 30% were defined as mild, moderate, and severe, respectively [7]. eGFR was estimated using the equation of Chronic Kidney Disease Epidemiology Collaboration (CKD-EPI). Patients with eGFR ≥ 90 mL/min/1.73 m^2^ were grouped into the normal renal function group (RN), whereas those with eGFR < 90 mL/min/1.73 m^2^ were included in the renal dysfunction group (RD) [21].

### Statistical analysis

Statistical analysis was performed using IBM SPSS Statistics 23.0 (IBM, Armonk, NY, USA). Quantitative data with a normal distribution were expressed as the mean and standard deviation and analyzed using *t*-tests. Quantitative data with a non-normal distribution were presented as the median and interquartile range and assessed by the Mann–Whitney U test. Numerical data were compared using the χ^2^ test or Fisher’s exact probability test. To balance baseline characteristics between RN and RD groups, propensity score matching (PSM) was employed, utilizing the nearest neighbor matching method with a caliper value of 0.03. A standardized mean difference (SMD) < 0.1 was considered indicative of well-balanced groups. Kaplan–Meier plots were used to present the probability of hematological side effects in relation to the duration of linezolid treatment. *P* < 0.05 was considered significant.

## Results

### **Baseline characteristics**,** linezolid concentrations**,** and hematological toxicity before propensity score matching**

In total, 320 elderly patients were included, of whom 66 (20.6%) were included in the RN group and 254 (79.4%) were included in the RD group. There were significant differences between the two groups regarding age, the duration of linezolid treatment, CCI, and the SOFA score (all *P* < 0.05, Table 1).

**Table 1:** Baseline characteristics, linezolid concentrations, and hematological toxicity before propensity score matching

Characteristics					All patients (*n* = 320)	Groups		*P*
						RN group	RD group	
						eGFR ≥ 90(*n* = 66)	eGFR < 90(*n* = 254)	
Age, years, x±s					89.0 ± 9.9	81.5 ± 10.5	90.9 ± 8.9	< 0.0001^§^
Age, N (%)								< 0.0001^*^
65–80					57(17.8)	29(43.9)	28(11.0)	
81–90					79(24.7)	22(33.3)	57(22.4)	
>90					184(57.5)	15(22.7)	169(66.5)	
Gender, male, N (%)					267(83.4)	56(84.8)	211(83.1)	0.729^*^
Weight, kg, x±s					61.75±11.3	60.5±10.0	62.1±11.6	0.130^§^
BMI, kg/m^2^, x±s					21.6±3.1	20.1±3.5	22.8±3.3	0.402^§^
Dose regimen, mg/kg, x±s					20.1±3.6	20.3±3.1	20.0±3.7	0.130^§^
Duration, days, median (IQR)					10[7,13]	11[8,14]	10[7,12.5]	0.021^#^
Duration ≥ 12 days, N (%)					115(36.1)	32(48.5)	83(32.8)	0.018^*^
Underlying disease, N (%)								
COPD					241(75.3)	52(78.8)	189(74.4)	0.462^*^
Hypertension					231(72.2)	36(54.5)	195(76.8)	< 0.0001^*^
Coronary Heart Disease					203(63.4)	39(59.1)	164(64.6)	0.411^*^
Atrial fibrillation					52(16.3)	13(19.7)	39(15.4)	0.394^*^
Diabetes mellitus					137(42.8)	43(65.2)	140(55.1)	0.142^*^
Neurological disease					130(40.6)	26(39.4)	104(40.9)	0.819^*^
Malignancy					76(23.8)	53(80.3)	191(75.2)	0.422^*^
CCI, median (IQR)					5[4,7]	5[4,6]	6[4,8]	0.002^#^
Invasive ventilation, N (%)					70(21.9)	11(16.7)	59(23.2)	0.251^*^
Septic shock, N (%)					67(20.9)	12(18.2)	55(21.7)	0.537^*^
Infection sites, N (%)								0.331^*^
Pulmonary infection					291(90.9)	58(87.9)	233(91.7)	
Others					29(9.1)	8(12.1)	21(8.3)	
Laboratory findings								
Albumin, g/L, x±s					34.1±5.0	34.2±5.2	34.0±5.0	0.751^§^
Creatinine, µmol/L, median (IQR)					85[60, 128]	44[38,56]	99[77,145]	< 0.0001^#^
eGFR, ml/min/1.73m^2^,median (IQR)					64[40,86]	98[94, 105]	53[34, 75]	< 0.0001^#^
Bilirubin, µmol/L, median (IQR)					12.0[7.8, 18.5]	11.8[7.3, 17.8]	12.1[8.0, 18.9]	0.655^#^
ALT, U/L, median (IQR)					18.0[10.6, 35.3]	19.2[12.7,43.7]	17.5[9.7, 34.5]	0.100^#^
Baseline Platelet,10^9^/L, median (IQR)					192[150, 236]	206[159, 261]	189[148, 231]	0.081^#^
Baseline Hemoglobin, g/L, median (IQR)					111[99, 124]	107[95, 123]	112[88, 154]	0.225^§^
Combination of antibiotics, N (%)								
Carbapenems					223(69.7)	45(68.2)	178(70.1)	0.765^*^
Cephalosporin					97(30.3)	21(31.8)	76(29.9)	0.765^*^
Antifungal drug					93(29.1)	18(27.3)	75(29.5)	0.719^*^
SOFA, median (IQR)					4[2,7]	3[1,6]	4[3,7]	0.001^#^
30-day mortality, N (%)					22(6.9)	3(4.5)	19(7.5)	0.440^*^
C_min, 3−5d_, mg/L, median (IQR)					21.7[12.8, 34.4]	12.3[6.7, 20.4]	24.4[15.6, 36.5]	< 0.0001^#^
C_min,6−10d_, mg/L, median (IQR)					24.9[15.3, 36.1]	15.3[9.9, 23.8]	27.7[17.7, 40.7]	< 0.0001^#^
C_ave_, mg/L, median (IQR)					23.1[14.6, 34.9]	13.2[8.1, 22.3]	26.9[16.9, 37.8]	< 0.0001^#^
C_ave_, mg/L, N (%)								< 0.0001^*^
					6(1.9)	5(7.6)	1(0.4)	
2–8					37(11.6)	13(19.7)	24(9.4)	
8–16					53(16.6)	20(30.3)	33(13.0)	
16–32					120(37.5)	22(33.3)	98(38.6)	
> 32					104(32.5)	6(9.1)	98(38.6)	
Thrombocytopenia, N (%)					221(69.1)	31(47.0)	192(75.6)	< 0.0001^*^
Mild					91(28.4)	15(22.7)	76(29.9)	
Moderate					79(24.7)	12(18.2)	67(26.4)	
Severe					53(16.6)	4(6.1)	49(19.3)	
Anemia, N (%)					193(60.3)	30(45.5)	163(64.2)	0.006^*^
Mild					99(30.9)	16(24.2)	83(32.7)	
Moderate					64(20.0)	11(16.7)	53(20.9)	
Severe					30(9.4)	3(4.5)	27(10.6)	

*RN *normal renal function, *RD *renal dysfunction, *BMI *body mass index, *COPD *chronic obstructive pulmonary disease, *CCI *charlson comorbidity index,* ALT *alanine aminotransferase, *eGFR *estimated glomerular filtration rate, *SOFA *sequential organ failure assessment

^*^Chi-square test

^*§*^t test

^*#*^Mann-Whitney U test

C_min,3−5d_, C_min,6−10d_, and C_ave_ were significantly lower in the RN group than in the RD group (all *P* < 0.0001, Table 1). The incidence of LAT and linezolid-associated anemia (LAA) was significantly lower in the RN group than in the RD group (both *P* < 0.05, Table 1).

### **Baseline characteristics**,** linezolid concentrations**,** and hematological toxicity after propensity score matching**

**Fig. 2:**
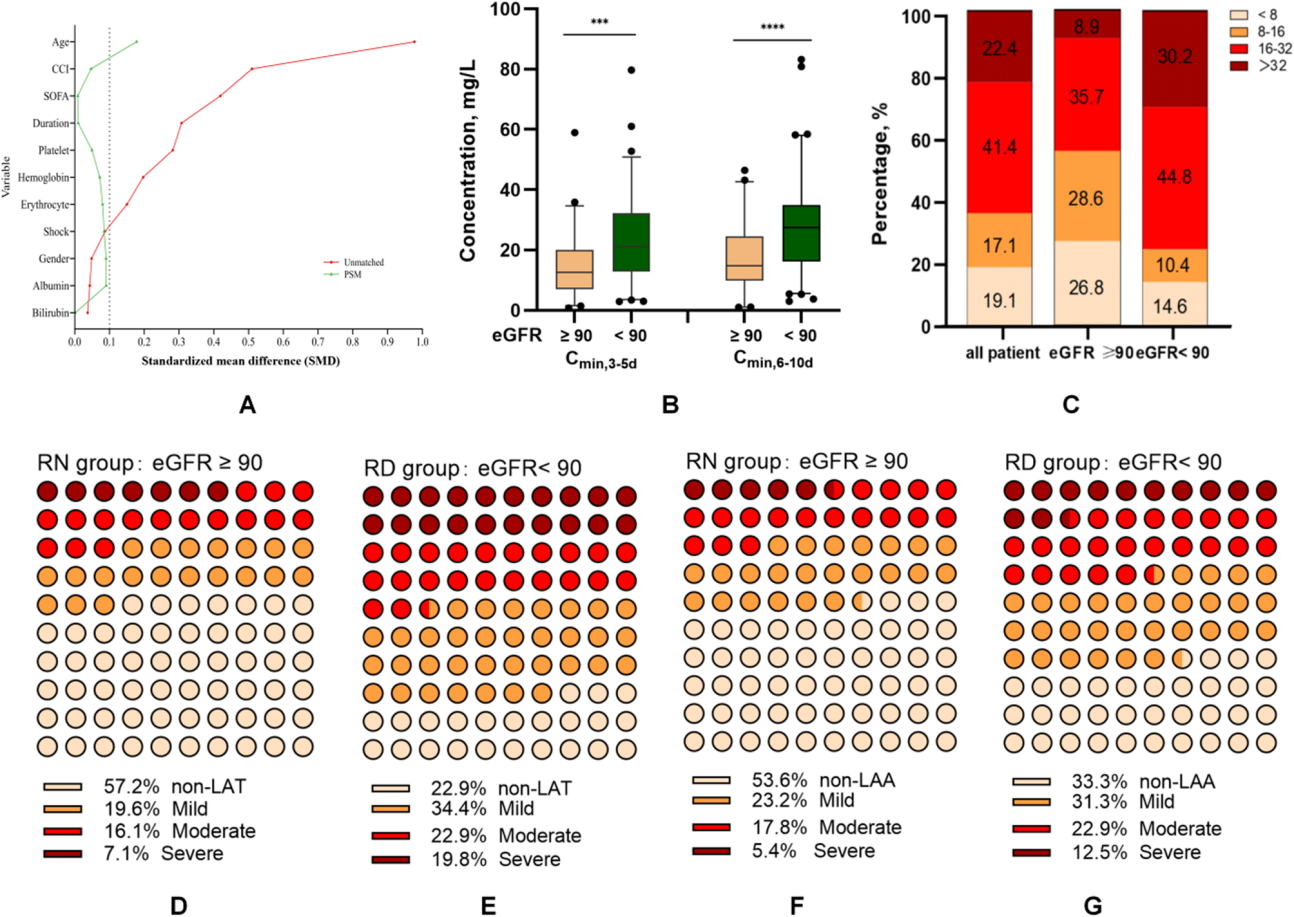
Effects of renal function on linezolid C_min_ and hematological toxicity. **A** Baseline characteristics before and after propensity score matching. **B** C_min_ at 3–5 and 6–10 days in patients with normal and impaired renal function. **C** Distribution of C_min_ in patients with normal and impaired renal function. **D** Distribution of linezolid-associated thrombocytopenia (LAT) in normal renal function (RN) group. **E** Distribution of LAT in renal dysfunction (RD) group. **F** Distribution of linezolid-associated anemia (LAA) in normal renal function (RN) group. **G** Distribution of LAA in renal dysfunction (RD) group

In total, 152 patients were enrolled after propensity score matching, including 56 (36.8%) patients in the RN group and 96 (63.2%) patients in the RD group. There were no differences in sex, age, the duration of linezolid treatment, CCI, SOFA scores, combination of antibiotics, and laboratory findings between the two groups (all *P* > 0.05, Fig. 2A).

C_min,3−5d_ was 12.5 [6.9, 20.1] mg/L in the RN group, versus 21.2 [12.9, 32.3] mg/L in the RD group (*P* < 0.0001, Table 2; Fig. 2B). C_min,6−10d_ was 14.8 [9.9, 24.5] mg/L in the RN group, versus 27.4 [16.2, 34.9] mg/L in the RD group (*P* < 0.0001, Table 2; Fig. 2B). The distributions of linezolid C_min_ were significantly different between the two groups (*P* < 0.0001, Table 2; Fig. 2C).

**Table 2:** Baseline characteristics, linezolid concentrations, and hematological toxicity after propensity score matching

Characteristics							All patients (*n* = 152)	Groups		*P*
								RN group	RD group	
								eGFR ≥ 90(*n* = 56)	eGFR < 90(*n* = 96)	
Age, years, x±s							84.1±10.3	82.9±10.0	84.7±10.5	0.295^§^
Age, N (%)										0.254^*^
65–80							48(31.6)	22(39.3)	26(27.1)	
81–90							54(35.5)	19(33.9)	35(36.45)	
>90							50(32.9)	15(26.8)	35(36.45)	
Gender, male, N (%)							130(85.5)	49(87.5)	81(84.4)	0.597^*^
Weight, kg, x±s							61.5±11.0	60.5±10.0	62.0±11.2	0.417^§^
BMI, kg/m^2^, x±s							22.1±3.2	21.8±3.3	22.3±3.4	0.393^§^
Regimen, mg/kg, x±s							20.1±3.6	20.2±3.1	20.0±3.9	0.703^§^
Duration, days, median (IQR)							11[8,14]	11[8,14]	11[8,13.75]	0.843^#^
Duration ≥ 12 days, N (%)							69(45.4)	27(48.2)	42(43.8)	0.616^*^
CCI, median (IQR)							5[3.25,6]	5[4,6]	4.5[3,6]	0.435^#^
Septic shock, N (%)							36(23.7)	12(21.4)	24(25.0)	0.617^*^
Laboratory findings										
Albumin, g/L, x±s							34.1±5.0	34.4±5.1	34.0±5.0	0.590^§^
Creatinine, µmol/L, median (IQR)							72[48,99]	43[37,55]	92[77,128]	< 0.0001^#^
eGFR, ml/min/1.73m^2^,median (IQR)							82[53,95]	97[94,105]	62[47,78]	< 0.0001^#^
Bilirubin, µmol/L, median (IQR)							12.4[7.8,18.7]	13.3[7.70,18.5]	12.3[8.2,18.9]	0.922^#^
ALT, U/L, median (IQR)							18.7[11.0,40.1]	18.3[12.1,49.2]	19.4[10.9,39.0]	0.525^#^
Platelet,10^9^/L, median (IQR)										
Baseline							197[156,244]	191[159,247]	200[154,243]	0.938^#^
withdrawal							130[98, 176]	145[102, 214]	121[95, 162]	0.014^#^
after withdrawal							111[78, 173]	162[89, 231]	102[74, 147]	0.002^#^
Decreases in counts							77[28,128]	64[3,107]	86[48, 132]	0.004^#^
Decreases in platelet percentages, %, median (IQR)							40.3[18.5, 58.5]	26.0[3.6, 46.6]	45.9[30.8, 65.6]	< 0.0001^#^
Hemoglobin, g/L, median (IQR)										
Baseline							108[97, 122]	108[98, 126]	108[97, 121]	0.668^#^
withdrawal							98[86, 111]	104[87, 115]	95[82, 109]	0.124^#^
after withdrawal							95[81, 109]	100[81, 113]	92[79, 105]	0.203^#^
Decreases in counts							14[4, 23]	10[2, 20]	16[6, 28]	0.049^#^
Decreases in hemoglobin percentages, %, median (IQR)							11.9[3.7, 20.9]	6.7[1.4, 17.6]	14.3[6.3, 23.2]	0.029^#^
Combination of antibiotics, N (%)										
Carbapenems							107(70.4)	39(69.6)	68(70.8)	0.877^*^
Cephalosporin							45(29.6)	17(30.4)	28(29.2)	0.877^*^
Antifungal drug							46(30.3)	15(26.8)	31(32.3)	0.476^*^
SOFA, median (IQR)							3[2,6]	3[2,6.75]	3[2,6]	0.983^#^
30-day mortality, N (%)							6(3.9)	2(3.6)	4(4.2)	0.856^*^
C_min, 3−5d_, mg/L, median (IQR)							17.2[8.2,29.4]	12.5[6.9,20.1]	21.2[12.9,32.3]	0.001^#^
C_min, 6−10d_, mg/L, median (IQR)							23.8[11.9,33.3]	14.8[9.9,24.5]	27.4[16.2,34.9]	< 0.0001^#^
C_ave_, mg/L, median (IQR)							20.9[10.9,30.0]	13.4[7.9,22.6]	24.7[15.8,34.5]	< 0.0001^#^
C_ave_, mg/L, N (%)										< 0.0001^*^
							4(2.6)	4(7.1)	0	
2–8							25(16.5)	11(19.7)	14(14.6)	
8–16							26(17.1)	16(28.6)	10(10.4)	
16–32							63(41.4)	20(35.7)	43(44.8)	
> 32							34(22.4)	5(8.9)	29(30.2)	
Thrombocytopenia, N (%)							99(65.1)	24(42.8)	74(77.1)	< 0.0001^*^
Mild							45(29.6)	11(19.6)	33(34.4)	
Moderate							31(20.4)	9(16.1)	22(22.9)	
Severe							23(15.1)	4(7.1)	19(19.8)	
Anemia, N (%)							90(59.2)	26(46.4)	64(66.7)	0.014^*^
Mild							43(28.3)	13(23.2)	30(31.3)	
Moderate							32(21.1)	10(17.8)	22(22.9)	
Severe							15(9.9)	3(5.4)	12(12.5)	

*RN *normal renal function, *RD *renal dysfunction, *BMI *body mass index, *COPD *chronic obstructive pulmonary disease,* CCI *charlson comorbidity index, *ALT *alanine aminotransferase, *eGFR *estimated glomerular filtration rate, *SOFA *sequential organ failure assessment

^*^Chi-square test

^§^t test

^#^Mann-Whitney U test

LAT occurred in 24 (42.8%) patients in the RN group, including 11 (19.6%), 9 (16.1%), and 4 (7.1%) patients in mild, moderate, and severe LAT, respectively, compared with 74 (77.1%) patients in the RD group, including 33 (34.4%), 22 (22.9%), and 19 (19.8%) patients with mild, moderate, and severe LAT, respectively (*P* < 0.0001, Table 2; Fig. 2D-E). LAA occurred in 26 (46.4%) patients in the RN group, including 13 (23.2%), 10 (17.8%), and 3 (5.4%) patients with mild, moderate, and severe LAA, respectively. In the RD group, 64 (66.7%) patients developed LAA, of whom 30 (31.3%), 22 (22.9%), and 12 (12.5%) patients had mild, moderate, and severe LAA, respectively. The difference between the two groups was significant (*P* = 0.014, Table 2; Fig. 2F-G).

### Effects of renal function on linezolid concentrations and hematological toxicity

**Fig. 3:**
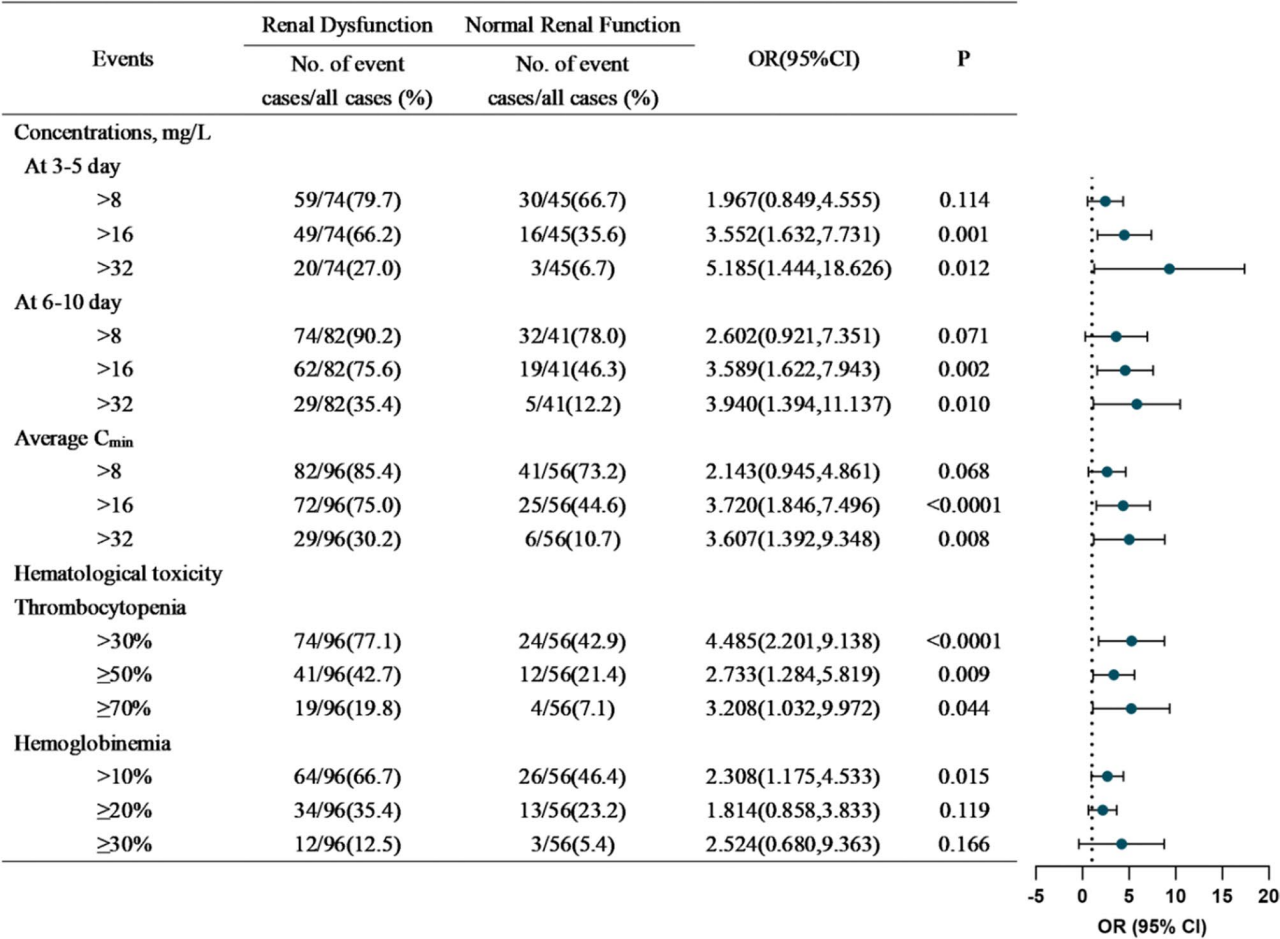
Effect of renal function on the distribution of linezolid C_min_ and the grading of hematological toxicity

Renal dysfunction was associated with C_min,3−5d_ >16 mg/L (odds ratio [OR] = 3.552, 95% confidence interval [CI] = 1.632–7.731, *P* = 0.001, Fig. 3), C_min,3−5d_ >32 mg/L (OR = 5.185, 95% CI = 1.444–18.626, *P* = 0.012, Fig. 3), C_min,6−10d_ >16 mg/L (OR = 3.589, 95% CI = 1.622–7.943, *P* = 0.002, Fig. 3), C_min,6−10d_ >32 mg/L (OR = 3.940, 95% CI = 1.394–11.137, *P* = 0.010, Fig. 3), C_ave_ >16 mg/L (OR = 3.720, 95% CI = 1.846–7.496, *P* < 0.0001, Fig. 3), and C_ave_ >32 mg/L (OR = 3.607, 95% CI = 1.392–9.348, *P* = 0.008, Fig. 3). There was no significant difference in the incidence of C_min_ >8 mg/L between the RN and RD groups (*P* > 0.05, Fig. 3).

Renal dysfunction was associated with decreases in the platelet count of > 30% (OR = 4.485, 95% CI = 2.201–9.138, *P* < 0.0001, Fig. 3), ≥ 50% (OR = 2.733, 95% CI = 1.284–5.819, *P* = 0.009, Fig. 3), and ≥ 70% (OR = 3.208, 95% CI = 1.032–9.972, *P* = 0.044, Fig. 3). Renal dysfunction was associated with a decrease in the hemoglobin level of > 10% (OR = 2.308, 95% CI = 1.175–4.533, *P* = 0.015, Fig. 3).

### Effects of renal function on hematological indices

**Fig. 4:**
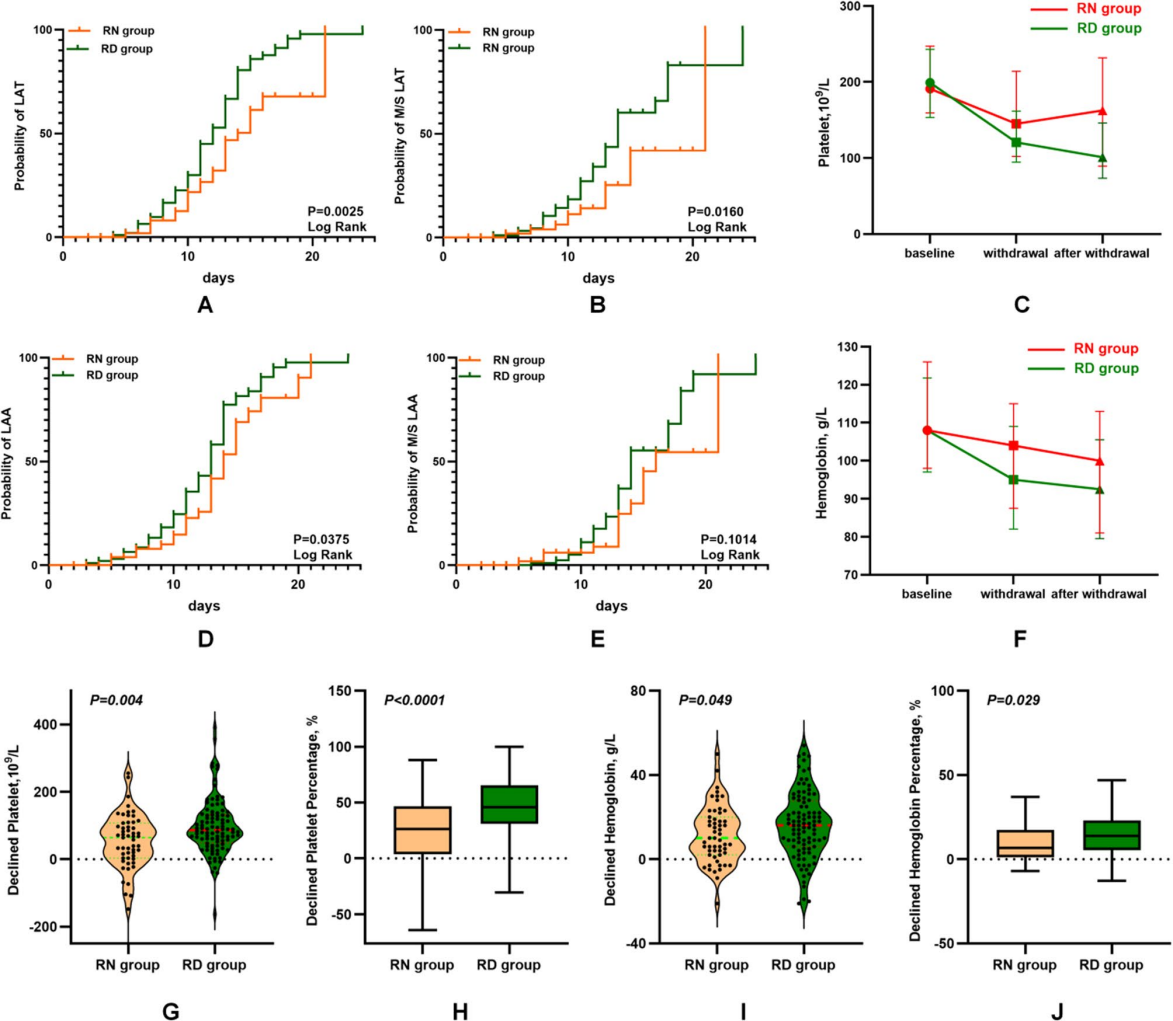
Effects of renal function on hematological indices. **A** The Kaplan–Meier curve displaying the probability of LAT in RN and RD groups. **B** The Kaplan–Meier curve displaying the probability of moderate-to-severe (M/S) LAT in RN and RD groups. **C** Trend chart of platelet counts in RN and RD groups. **D** The Kaplan–Meier curve displaying the incidence of LAA in RN and RD groups.** E** The Kaplan–Meier curve displaying the incidence of M/S LAA in RN and RD groups. **F** Trend chart of hemoglobin levels in RN and RD groups.** G** Decreases in platelet counts in RN and RD groups. **H** Decreases in platelet percentages in RN and RD groups. **I** Decreases in hemoglobin levels in RN and RD groups.** J** Decreases in hemoglobin percentages in RN and RD groups

The incidence of LAT and moderate-to-severe (M/S) LAT increased gradually as the duration of linezolid, and the incidence was significantly higher in RD group than in RN group (*P* = 0.0025 and 0.0160, Fig. 4A-B). Decreases in platelet counts were shown in Fig. 4C.

The incidence of LAA was significantly higher in RD group than in RN group (*P* = 0.0375, Fig. 4D), whereas no difference was found in incidence of M/S LAA between the two groups (*P* = 0.1014, Fig. 4E). Decreases in hemoglobin levels were shown in Fig. 4F.

The decreases in platelet counts and percentages were significantly higher in RD group than in RN group (*P* = 0.004 and < 0.0001, Fig. 4G–H), and the decreases in hemoglobin levels and percentages were significantly higher in RD group than in RN group (*P* = 0.049 and = 0.029, Fig. 4I–J).

## Discussion

The study included 320 elderly patients who received linezolid, and after propensity score matching, 152 patients were enrolled. We found that the C_min_ of linezolid in patients with renal dysfunction was approximately 2-fold higher than that in patients with normal renal function, and the risks of LAT and LAA were approximately 4.5-fold and 2.5-fold higher in patients with renal dysfunction, revealing that renal dysfunction significantly increased linezolid C_min_ and linezolid-associated hematological toxicity in elderly patients. Given the significant impact of renal function on linezolid C_min_ and hematological indices in elderly patients, we also recommended that elderly patients with renal dysfunction should receive a reduced dose of linezolid.

In this study, renal dysfunction significantly increased linezolid C_min_ in elderly patients, possibly because 30% of the linezolid dose is eliminated unchanged via the kidneys [22]. Although it has been reported that linezolid clearance is not affected by renal function [23], a series of studies identified renal function as an important factor affecting linezolid C_min_ [3, 8–13]. Cattaneo et al. [12] found a significant positive linear correlation between serum creatinine and linezolid C_min_ (*R* = 0.511, *P* < 0.01) and recommended reducing the dose of linezolid in patients with renal impairment. Nukui et al. [13] reported that the C_min_ of linezolid was significantly increased in patients with creatinine clearance < 60 mL/min (14.7 mg/L vs. 4.8 mg/L) and recommended that the linezolid dose should be adjusted to 600 mg/day in patients with renal dysfunction. We also found that the C_min_ of linezolid was significantly increased in patients with renal dysfunction by approximately 2-fold versus that in patients with normal renal function. In particular, the risk of C_min_ >16 mg/L and the C_min_ >32 mg/L were increased by approximately 3–5 folds. Given the significant impact of renal function on linezolid C_min_ in elderly patients, we also recommended that patients with renal dysfunction should receive a reduced dose of linezolid. Therefore, there was an urgent need for a population pharmacokinetics (PopPK) model of linezolid in elderly patients to guide individualized dose regimens.

Consistent with previous studies [14–19], we found that the incidence of linezolid-associated hematological toxicity was significantly increased in patients with renal dysfunction, which might be attributable to overexposure to linezolid. However, in addition to linezolid overexposure, renal function can independently influence hematological toxicity. Liu et al. [7] reported that eGFR < 60 mL/min/1.73 m^2^ was an independent risk factor for M/S LAT, and its risk was approximately 2-fold higher in this group than in the control group. Crass et al. [18] found that the incidence of thrombocytopenia was significantly increased in patients with renal dysfunction (42.9% vs. 16.8%), and renal dysfunction could independently predict thrombocytopenia suggesting that although linezolid C_min_ can be controlled within the safe and effective range by TDM, elderly patients with renal dysfunction require close monitoring of hematologic indices to avoid serious side effects. In addition, the majority of linezolid is metabolized via oxidation of its morpholino ring to create two metabolites (PNU-142586 and PNU-142300), and it is unclear whether these two metabolites induce hematological toxicity [22]. However, Xu et al. [19] reported that the concentration of PNU142300 was significantly elevated in patients with renal dysfunction and was independently associated with thrombocytopenia.

This study had some limitations. Firstly, as a non-intervention study, patients did not receive dose adjustment based on C_min_ even though clinicians were aware of overexposure. Secondly, although we have used the most common definitions of thrombocytopenia and anemia, the current definitions are not uniform, and the mechanism of linezolid-associated hematological toxicity was not completely clear. Thirdly, although a series of western studies are similar to the results of this study [8–14], the C_min_ of linezolid in elderly Chinese patients is significantly higher [5, 7, 13]. Therefore, the origin of the population may affect the C_min_ and hematotoxicity of linezolid. However, this study did not further clarify this issue. Fourthly, we are currently unable to recommend reduced dose regimens. A PopPK model of linezolid for elderly patients is required to optimize individualized dosing regimens.

## Conclusion

In this propensity-matched cohort analysis, we found that renal function significantly influenced linezolid C_min_ and hematological toxicity in the elderly population. Elderly patients with renal dysfunction should receive a reduced dose of linezolid. A PopPK model of linezolid for elderly patients is required to optimize individualized dosing regimens in the future.

## Data Availability

The data used and/or analyzed during the current study are available from the corresponding author on reasonable request.
